# From Smoothies to Dialysis: The Impact of Oxalate Nephropathy

**DOI:** 10.7759/cureus.67409

**Published:** 2024-08-21

**Authors:** Mohammed Samra, Isha Gupta

**Affiliations:** 1 Health Sciences, University of Debrecen, Debrecen, HUN; 2 Nephrology, Middletown Medical, Middletown, USA; 3 Nephrology, Garnet Health Medical Center, Middletown, USA; 4 Internal Medicine/Nephrology, Touro College of Osteopathic Medicine, Middletown, USA

**Keywords:** chronic hemodialysis, oxalate crystals, kidney biopsy, acute kidney injury, end stage renal disease, secondary oxalate nephropathy

## Abstract

Oxalate nephropathy is a rare cause of acute kidney injury that can lead to end-stage renal disease. This case report describes a 54-year-old male with type 2 diabetes mellitus and chronic kidney disease who presented for a routine clinic follow-up. Laboratory tests revealed significant deterioration in renal function with an unrevealing history and symptoms suggestive of the process. Initial investigations for worsening renal function were inconclusive, prompting a renal biopsy that confirmed acute tubular injury with abundant calcium oxalate deposits. Further investigation into dietary history revealed that the patient regularly consumed high-oxalate foods, such as spinach and kale smoothies, under the impression they were beneficial for his diabetes. Despite the initiation of hemodialysis, the patient did not recover renal function and remains dialysis-dependent. This case underscores the need for a high index of suspicion for oxalate nephropathy in chronic kidney disease patients presenting with unexplained acute kidney injury. Diagnosis is confirmed through renal biopsy and should be considered in patients with relevant dietary histories.

## Introduction

Oxalate nephropathy (ON) is a disease that affects the renal tubules and is characterized by the deposition of calcium oxalate crystals in the renal parenchyma [1,2]. ON is a rare yet significant cause of acute kidney injury (AKI), which most often progresses to end-stage renal disease (ESRD) [3]. ON can arise either from primary hyperoxaluria or from secondary causes of hyperoxaluria [1].

Primary hyperoxaluria is a broad group of genetic disorders that involve enzymatic defects in the glyoxylate pathway, which leads to impaired oxalate metabolism and subsequent crystallization within the renal tubules [4].

Secondary hyperoxaluria is a more common cause of ON but is often underrecognized [5]. Many factors are associated with its development [6]. The factors include but are not limited to increased intestinal absorption of oxalate, nutritional deficiencies, reduced fluid intake, impaired renal excretion, and a high oxalate diet [7,8].

The clinical presentation of ON varies between patients, and its diagnostic criteria remain insufficiently defined, underscoring the importance of increased clinical awareness for effective recognition and management to prevent irreversible renal damage [9].

Here, we report a case of a dialysis requiring ESRD caused by biopsy-proven acute ON.

## Case presentation

A 54-year-old male with a history of type 2 diabetes mellitus (T2DM) with diabetic retinopathy and chronic kidney disease (CKD) stage 3A was seen in the nephrology clinic for a follow-up regarding abnormal kidney function tests. Repeat laboratory testing performed at the clinic showed blood urea nitrogen (BUN) 125 mg/dl (baseline 37 mg/dl), creatinine 8.6 mg/dl (baseline 1.7 mg/dl), estimated glomerular filtration rate (eGFR) 6.8 ml/min/1.73 m^2^ (baseline 45 ml/min/1.73 m^2^) (Table 1). His glycated hemoglobin (HbA1c) was at 5%, similar to previous readings. On physical examination, no significant findings were noted. The patient reported feeling healthy, denied any episodes of diarrhea or vomiting, and confirmed no use of new medications or non-steroidal anti-inflammatory drugs (NSAIDs). Subsequently, the patient was admitted to the hospital and an extensive renal workup was initiated for evaluation of his AKI on CKD.

**Table 1:** Lab values of the patient prior to admission BUN: blood urea nitrogen; eGFR: estimated glomerular filtration rate; PTH: parathyroid hormone; HbA1c: glycated haemoglobin

Test	Result	Baseline (3 months ago)	Normal Hospital Range
BUN	125 mg/dL	37 mg/dL	7-20 mg/dL
Creatinine	8.6 mg/dL	1.7 mg/dL	0.6-1.3 mg/d
eGFR	6.8 mL/min/1.73 m²	45 mL/min/1.73 m²	>60 mL/min/1.73 m²
Albumin Creatinine Ratio	>300 mg/g	<30 mg/g	<30 mg/g
Intact PTH	724 pg/mL	150 pg/mL	10-65 pg/mL
Calcium	7.7 mg/dL	9 mg/dL	8.5-10.2 mg/dL
Phosphorus	7.8 mg/dL	3.5 mg/dL	2.5-4.5 mg/dL
HbA1c	5%	5%	4-6%
Hemoglobin	9.9 g/dL	13 g/dL	12-16 g/dL
Hematocrit	30%	40%	36-48%
Platelet	125 x 10³/µL	128 x 10³/µL	150-450 x 10³/µL

**Figure 1:**
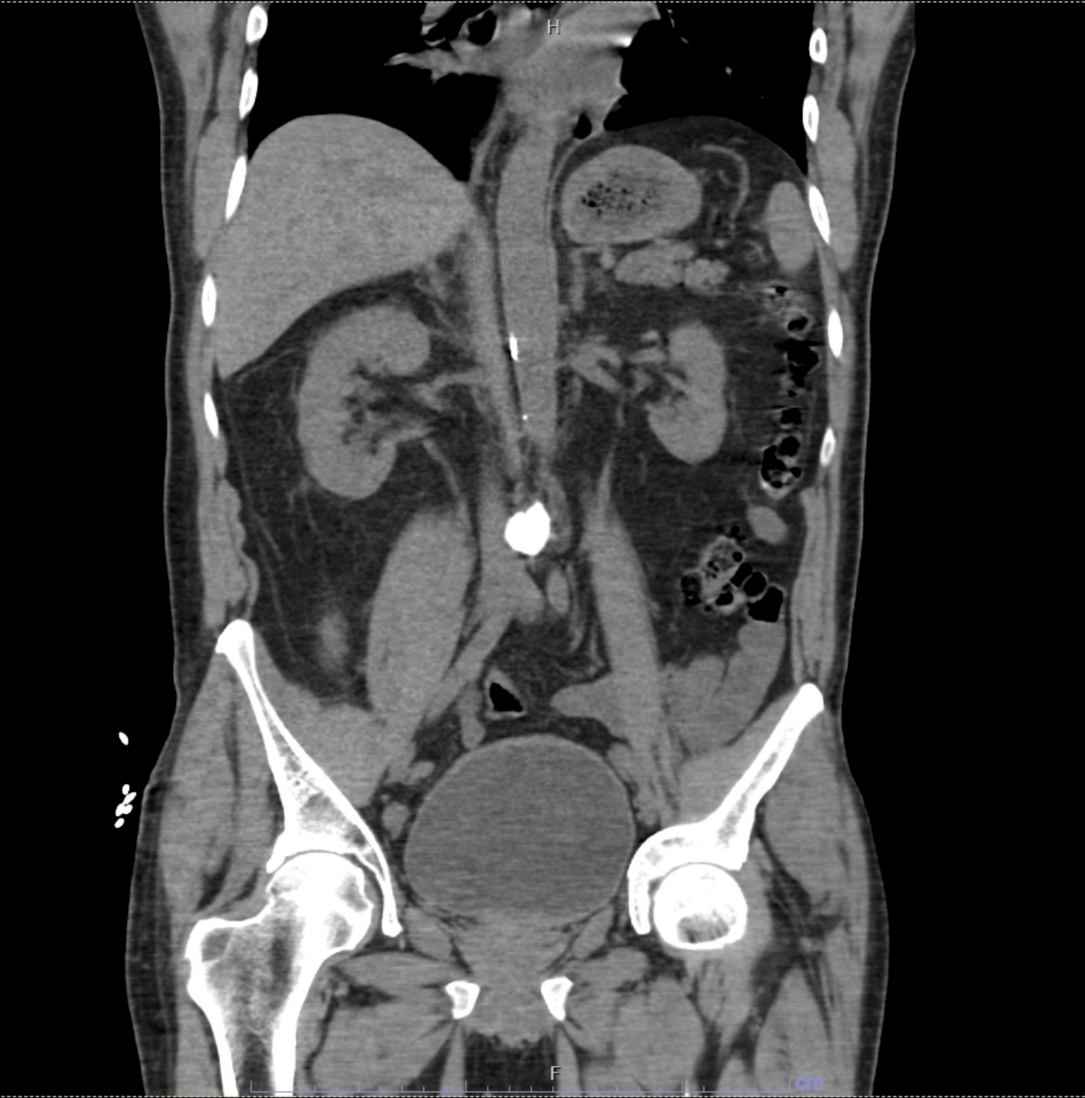
Coronal view of CT abdomen pelvis without IV contrast

The renal workup showed normal C3 and C4 levels and a negative antineutrophilic cytoplasmic antibody (ANCA) panel, anti-glomerular basement membrane (anti-GBM), and anti-myeloperoxidase (anti-MPO). A serological workup for hepatitis B virus, hepatitis C virus, and HIV was performed, and the results were negative. Further urine and serum electrophoresis showed no monoclonal gammopathy. In addition, noncontrast CT did not show any abnormalities (Figure 1). As these investigations failed to provide a definitive cause for the AKI, a renal biopsy was performed on Day 3 of hospital admission.

**Figure 2:**
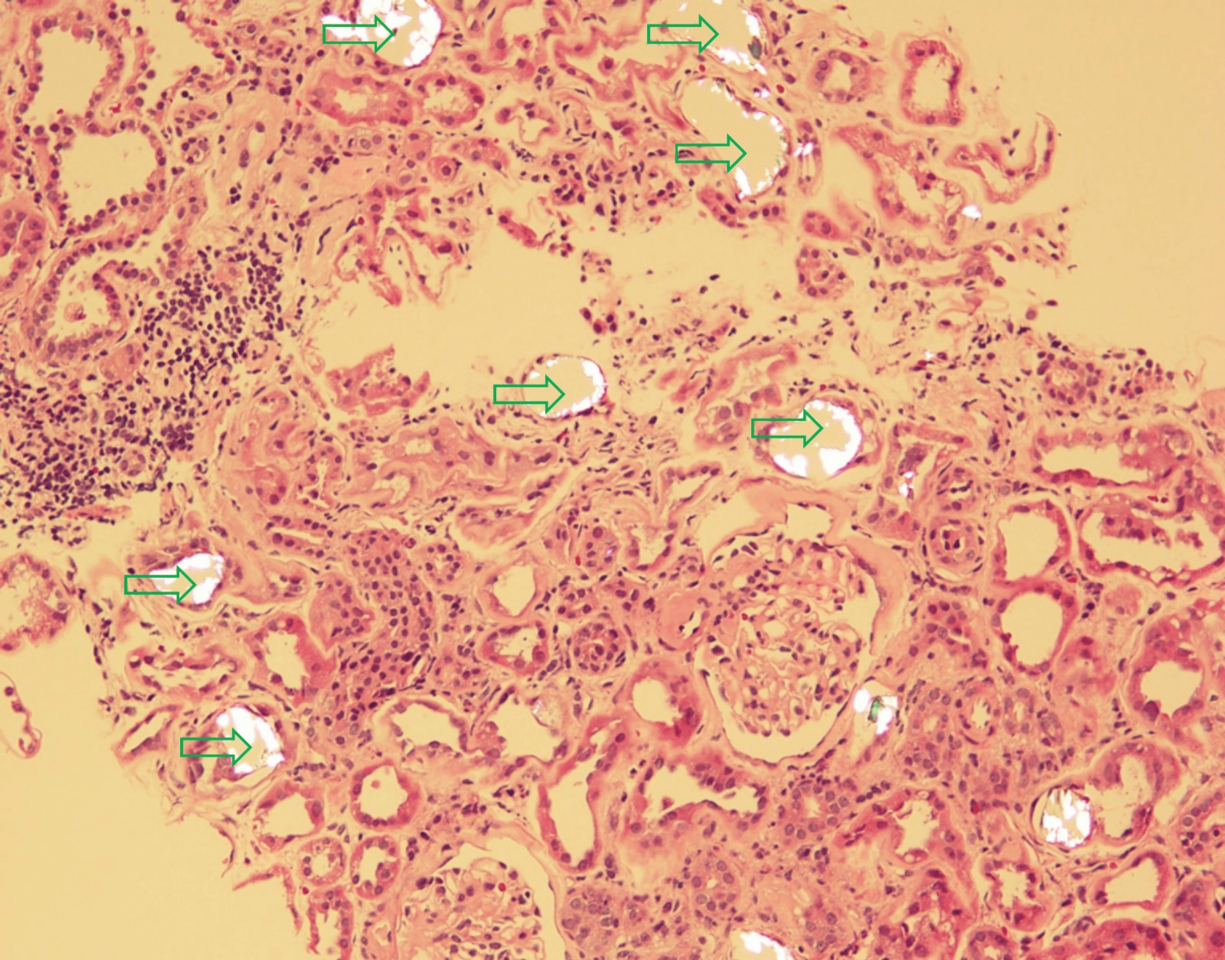
Renal histology under light microscopy at 30x magnification revealing abundant oxalate deposits Green arrows highlight calcium oxalate deposits, forming intraluminal translucent crystals.

On Day 4, there was no improvement in renal function and the patient was persistently azotaemic. Hence, it was decided that the patient would undergo hemodialysis. The biopsy report revealed abundant tubular calcium oxalate deposits (Figure 2). Electron microscopy revealed that numerous tubules contain calcium oxalate crystals along with PAS-positive hyaline casts. The interstitium contained interstitial inflammation composed of mononuclear cells. Approximately 40-50% of the renal cortex showed tubular atrophy and interstitial fibrosis. Arteries showed mild sclerosis and arterioles showed moderate sclerosis and hyalinosis. Diffuse flattening and loss of brush borders were also noted in the proximal tubular epithelium. Immunofluorescence microscopy was negative for paraprotein or immune complex deposition.

Based on these findings, a diagnosis was made of severe acute tubular injury with calcium oxalate deposits consistent with oxalate nephropathy superimposed upon moderate glomerulosclerosis, moderate tubular atrophy and interstitial fibrosis, and mild to moderate vascular sclerosis.

After reviewing the biopsy findings, a detailed dietary history was obtained from the patient’s sister. According to her, the patient used to daily consume a concoction of green leafy vegetables, which included spinach and kale, one orange, and a lemon. These foods are known to be high in oxalate, contributing significantly to dietary oxalate intake. In the absence of any other factors, this dietary habit could potentially explain the development of ON in the patient [10,11]. Unfortunately for the patient, his kidneys were not able to recover from this acute attack, and to date, he remains on hemodialysis.

## Discussion

This case highlights the debilitating nature of oxalate crystal deposition in tubules. This relatively young patient, who had been stable with an eGFR of 45 ml/min/m^2^, suddenly had an acute tubular injury that caused him to progress to ESRD and become hemodialysis dependent. According to the literature, the prognosis for ON is poor and most cases progress to chronic kidney failure [12]. Higher serum creatinine levels at presentation and tubular atrophy are associated with worse prognosis [13].

The underdiagnosis of this disease often delays early suspicion and accurate diagnosis [14]. Renal biopsy is the gold standard for diagnosis; however, 24-hour urine oxalate measurements could also provide an insight albeit it being rarely performed outside of the setting of nephrolithiasis [15]. An interesting point to be noted in this case is the patient’s diet. The patient’s sister unknowingly blended foods high in oxalate into a smoothie and incorporated this into the patient’s diet for daily consumption, believing it to be a healthy choice for a diabetic. Given the history of this patient’s CKD and diabetes history, this dietary practice might have hindered oxalate excretion, potentially contributing to ON [16]. Whether CKD itself predisposes individuals to reduced urinary oxalate excretion remains uncertain [17].

The patient received counseling on appropriate rehydration and the importance of taking calcium supplements. Additionally, he was advised to follow a low-fat, low-oxalate diet and to avoid foods rich in vitamin C [18,19].

## Conclusions

ON is a rare but critical disease that can rapidly progress to ESRD as depicted in this report. Our patient, who had suddenly deteriorated due to the long-term consumption of high-oxalate food, underscores the importance of carefully reviewing dietary history in CKD patients presenting with AKI. The elusiveness of the disease warrants heightened clinical awareness and timely diagnostic measures such as a renal biopsy. Early identification and intervention, including dietary modifications and patient education on oxalate-rich foods, are crucial in preventing irreversible renal damage and progression to ESRD. Further research is warranted to elucidate the interplay between CKD and oxalate metabolism, potentially paving the way for targeted preventive strategies and improved patient outcomes in this challenging condition.
